# The prevalence and predictors of feeding difficulties in children at self-feeding transition stage

**DOI:** 10.3389/fped.2023.1175927

**Published:** 2023-07-10

**Authors:** Meng Yan Tang, Xiao Mei Liu, Fan Yang

**Affiliations:** ^1^ Department of Child Health Care, West China Women’s and Children’s Hospital: Sichuan University West China Second University Hospital, Chengdu, China; ^2^ West China School of Nursing, Sichuan University, Chengdu, China; ^3^ Key Laboratory of Birth Defects and Related Diseases of Women and Children (Sichuan University), Ministry of Education, Chengdu, China

**Keywords:** feeding difficulties, prevalence, predictor, children, feeding practice

## Abstract

**Aim:**

To understand the prevalence of feeding difficulties (FD) in young children at self-feeding transition stage (6–24 months age), and the protective and risk predictors associated with FD are to be determined through this study.

**Methods:**

A cross-sectional study was conducted within 5 representative Women's and Children's hospitals in Chengdu, Southwest China. Children age 6–24 months who underwent routine child health care examination at outpatient and their parents were enrolled, while the Montreal Children's Hospital Feeding Scale which is validated was used to determine whether these children have FD.

**Results:**

A total of 1,211 subjects were enrolled in this survey, where 380 children were reported as FD with an prevalence of 31.4%. Adjusted binary logistic regression in the multivariate analysis showed 10 independent predictors of FD. Specifically there were 6 risk predictors: (1) frequent constipation (*OR* = 1.603, *CI* = 1.006–2.555) in CHILD sub-theme; (2) anxiety (*OR* = 4.322, *CI* = 3.074–6.079) and (3) indulgent parenting style (*OR* = 2.108, *CI* = 1.306–3.405) in PARENT sub-theme; (4) luring to eat (*OR* = 2.806, *CI* = 2.000–3.937), (5) forcing to eat (*OR* = 2.040, *CI* = 1.407–2.958), and (6) allowing playing during mealtime (*OR* = 2.023, *CI* = 1.435–2.853) in FEEDING PRACTICE sub-theme. The remaining 4 factors were protective predictors including (1) food preparing (*OR* = 0.586, *CI* = 0.385–0.891) in FOOD sub-theme; (2) observing hunger and satiety signals (*OR* = 0.667, *CI* = 0.457–0.974), (3) interacting with child during mealtime (*OR* = 0.505, *CI* = 0.308–0.828), as well as (4) providing exclusive tableware (*OR* = 0.370, *CI* = 0.191–0.719) in FEEDING PRACTICE sub-theme.

**Conclusions:**

There appeared to be an increasing trend of FD prevalence. Child health care clinicians and pediatricians are expected to attach more importance to FD in their daily work, and are obliged to provide parents with practical and effective preventive strategies highlighted in this study.

## Introduction

A suitable and balanced nutritional intake has been proved essential for both physical growth and neuropsychological development of young children. Aware that feeding is an integrated process involving complex parent-child interaction (1), parents tend to experience stress trying to keep an appropriate diet for young children. In recent decades, due to improved educational level of the populations, people have been paying more emphasis on child-raising, and more attention has been drawn to young children's feeding problems. Relevant literature has reported that up to 50% of parents claimed that their young children had various feeding problems, whose typical manifestations include poor appetite, food selectivity, sluggish eating, food refusal, prolonged dependence on liquid/soft food, poor chewing ability, temper tantrum, destructive behavior during mealtime, etc. These feeding problems are suggested to increase the risk of malnutrition and growth retardation, cause certain adverse impacts on children's growth and development (2, 3), or even threaten a far-reaching impact on their adulthood eating habits. Therefore, Feeding Difficulties (FD) is becoming a common and prominent issue to be tackled in the work of child health care and pediatric clinic.

In a broad sense, FD is an umbrella term which encompasses all spectrum of feeding problems, namely all that affect the process of supplying food for the child (4). But in practice, definitions of FD and its diagnostic criteria vary among different associations or experts based on their own perspectives and application convenience. In 1994, the Diagnostic and Statistical Manual of Mental Disorders (DSM-IV) proposed the concept of Feeding Disorder in Infancy and Early Childhood (FDIEC). While in 2013, DSM-V altered FDIEC to Avoidant Restrictive Food Intake Disorder (ARFID), defined as an eating or feeding disturbance in which children cannot maintain normal nutrition and exhibit food selectivity, poor appetite, or fear/anxiety about eating that is not related to cultural feeding practices. ARFID can be diagnosed when children fulfil at least one of the following four criteria: weight loss or poor growth, nutrient deficiency, dependence on oral or enteral supplements, and significant psychosocial dysfunction (5). More recently, a multi-disciplinary expert consensus in 2019 has put forward a unified diagnostic term for Feeding Difficulties—Pediatric Feeding Disorder (PFD). PFD is distinguished as a common clinical diagnosis which points out an inability to orally consume an age-appropriate diet, and should be diagnosed in the presence of dysfunction in at least one of four functional domains (medical, nutritional, skill and psychosocial) which persists for 3 months and beyond (6). Although both AFRID and PFD provide specific and detailed diagnostic criteria about FD, they are proved too strict and theoretical to be implemented in the clinic. Considering the utilizing convenience to identify and address FD, Kerzner has presented a functional and stepwise approach for clinicians to adopt when faced with children and parents undergoing FD (7). In Kerzner's studies (7–9), three principal categories of FD were defined as limited appetite, selective intake, and fear of feeding. 25%–50% of young children included in the studies were reported to have FD, but only 5%–10% needed intervention. Notably, certain red flags (i.e., warning signals) and practical feeding guidelines were strongly suggested by Kerzner in his proposal, which inspired us to carry out our research. Although not involved in Kerzner's studies, prevention of FD is believed by us to be of great significance for child health care clinicians and pediatricians, which involves understanding potential predictors of FD, and forming targeted preventive measures.

Self-feeding transition stage refers to a special period during which children transfer from being fed with liquid food to eating solid food on their own. According to the World Health Organization's recommendation that “from the age of 6 months, children should begin eating safe and adequate complementary foods while continuing to breastfeed for up to 2 years and beyond”, the age between 6 and 24 months is often considered as the self-feeding transition stage (10). At this stage, young children need to go through the conversion from being fed human milk to other types of milk, experience foods with different textures and multiple flavors, and learn dining rules as well as to use their own tableware. In this dynamic process of conversion, any problem linked with “child, food, parent, feeding practice” may lead to a high risk of developing FD for young children (11). In addition, previous studies have proved that food preferences and eating disorders in adulthood are significantly correlated to eating behavior problems in the first 2 years of life (12, 13). However, numerous current studies related to FD are mainly focused on preschool and school-age children (3–12 years of age), whereas only few studies have been aimed to understand the prevalence and predictors of FD in young children 6–24 months of age. Thus, we conducted this multi-center cross-sectional survey to understand the prevalence of FD in young children at the self-feeding transition stage, and independent predictors including both risk and protective factors are screened through our study. Ultimately, we hope the results and conclusions we represent here will be able to offer meaningful insights and guidance to child health care clinicians and pediatricians, so as to provide aids for young children and their parents faced with FD.

## Methods

### Study design

This is a multi-center cross-sectional study conducted in Chengdu—the regional development center of Sichuan province in southwestern China. Five research centers were recruited in this study, of which three representative university-affiliated maternal and child health hospitals were selected by convenience, and two district maternal and child health hospitals were selected randomly (selected by the random number generator from SPSS 22.0 statistical software) out of eleven districts of Chengdu.

• West China Women's and Children's Hospital: West China Second University Hospital, Sichuan University
• Sichuan Province Maternity and Child Health Care Hospital
• Chengdu Women's and Children's Central Hospital
• Qingbaijiang District of Chengdu Maternity and Child Health Care Hospital
• Longquan District of Chengdu Maternity and Child Health Care Hospital

### Samples and participants

We used PASS 15.0 software (NCSS, LLC, Kaysville, Utah, USA, ncss.com/software/pass.) to calculate our questionnaire sample size. The calculating model of confidence intervals for one proportion was run where confidence level (1−α) = 0.95, confidence interval width = 0.04, and the proportion was set as 0.214 according to a previous prevalence study in China (14). As a result, the sample size desired in this study was 1,664 questionnaires. Considering varied daily outpatient visits of each research center, we allocated 400 questionnaires to each of the three university-affiliated hospitals and 232 questionnaires each to two district hospitals. This multi-center cross-sectional study conducted a consecutive recruitment from August 2021 to May 2022. Two research nurses were employed to distribute questionnaires to parents who happened to be at the outpatient clinics of child health care in each research center, and results were collected on the spot once the questionnaires were filled in. Meanwhile, to ensure the quality of the study, well-trained research nurses were available to provide consultation and help when parents encountered any difficulty and confusion during the surveys. Children and their parents who underwent routine child health care examination at these five hospitals were enrolled in the study. The inclusion criteria requested that the parents were supposed to show a normal level of cognition and communication skills, capable of completing the questionnaire, and that their children were of 6–24 months of age (corrected gestational age should be adopted in the case of preterm children) who have been supplied with complementary food for at least 2 weeks. Parents who refused to participate in this study were excluded. All the participants signed an informed consent before filling their questionnaires, and ethical approval was obtained for our study from the Ethics Committee of Western China Women's and Children's Hospital of Sichuan University (West China Second University Hospital, Sichuan University).

### Measurements

The structured parent self-reported questionnaires involved in this study consisted of three parts. Firstly, there were demographic characteristics questionnaire including child age, caregiver, education of caregiver, parity, fetus number, maternal age, delivery, complication during pregnancy and household monthly income per capita. The remaining two parts included:

• Potential Predictors of FD Questionnaire: According to a Chinese expert Zhao's review of FD in infant, it is believed that the associated factors of FD during self-feeding transition stage mainly include children, food, parents and feeding practices (11). Therefore, this study designed a questionnaire to evaluate the potential associated factors of FD in children aged 6–24 months based on this viewpoint, and divided it into four sub themes as CHILD, FOOD, PARENT, and FEEDING PRACTICE. In addition, some of the items were designed based on the warning signs and specific practical methods mentioned by Dr. Kerzner's in his study on feeding difficulties (7–9). 8 factors were included in the CHILD sub-theme: gestational age, weight at birth, disease at birth, frequent constipation, frequent vomiting, frequent diarrhea, frequent food allergy and cow's milk protein allergy; 3 in FOOD: exclusive breastfeeding, time of introducing complementary food and food preparing; 4 in PARENT:anxiety, quarrels, parenting style [typically, parents choose a particular one out of four general parental feeding styles, which are responsive-responding to child's cues, controlling-overriding child's cues, indulgent-catering to child's desires, or neglectful-unaware of child's cues (9)] and time for parenting company; and 12 in FEEDING PRACTICE: observing hunger and satiety signals, training of self-feeding, luring to eat, forcing to eat, allowing playing during mealtime, interacting with child during mealtime, between-meal nibbles, meal time limit, providing exclusive tableware, providing fixed table and chair, quiet environment, and eating with parents. As can be seen, a total of 27 potential predictors were enrolled in this part of questionnaire.
• The Montreal Children's Hospital Feeding Scale: this scale with good reliability and validity was established by doctor M Ramsay to allow pediatricians and other health care professionals for a quick identification of FD in children from 6 months to 6 years of age (15). 14 parent-report items are graded based on a seven-point Likert scale. The total raw score is obtained by adding the scores for each item after reversing the scores of the 7 items graded from negative to positive. And the scoring sheet allows a quick conversion of raw scores into T-scores, which can then be classified as non-FD (below 61), mild (61–65), moderate (66–70) or severe (above 70). This scale was used in our study as a measurement of outcome indicators.

The questionnaire and scale used in this study has been translated into English and attached at the end of the manuscript, attached as Supplementary Appendix S1.

### Data analysis

We used SPSS 22.0 statistical software (IBM Corp, Chicago, IL, USA, www.ibm.com/legal/copytrade.shtml) to analyze the statistical data. First, descriptive statistics is used to analyze demographic data and FD prevalence which were described as “mean score ± standard deviation” or “number (percentage)”. Next, covariates were determined by *t*-test for continuous variables and chi-square test for categorical variables, to detect statistically significant differences of demographic baseline data between FD and Non-FD groups. Then, chi-square test was employed to select factors significantly associated with FD among 27 possible predictors under four sub-themes (the Fisher's Exact Test is required if the sample size is less than 5). Variables selected from univariate analysis were subsequently entered in a binary logistic regression model, where covariates were put in to adjust the model and categorical variables were processed as dummy variables. A *p*-value less than or equal to 0.05 was considered statistically significant.

## Results

### Demographic characteristics

A total of 1,211 valid questionnaires were returned with an effective rate of 72.78% (The effective rate of the five research centers are similar, with no significant difference between sites). Demographic characteristics are presented in Table 1. In comparing the baseline data between FD and non-FD groups, significant differences were detected in maternal age, complication during pregnancy and household monthly income per capita. Therefore, these were determined covariates that were later to be put in the logistic regression to adjust the model.

**Table 1:** Difference of demographic characteristics between FD and non-FD groups.

Variables	FD (*n* = 380)	Non-FD (*n* = 831)	*t*/*χ*^2^	*p*
Child age (month, mean ± std)	14.02 ± 5.45	13.68 ± 5.35	−1.011^a^	0.312
Child age (*n*, %)				
6–12 months	180 (47.4)	422 (50.8)		
13–18 months	126 (33.2)	258 (31.0)		
19–24 months	74 (19.4)	151 (18.2)	1.216^b^	0.544
Caregivers (*n*, %)				
Parents	241 (63.4)	569 (68.5)		
Grandparents	125 (32.9)	228 (27.4)		
Babysitters	14 (3.7)	34 (4.1)	3.768^b^	0.152
Education of caregivers (*n*, %)				
High school	142 (37.4)	268 (32.3)		
College or bachelor	218 (57.4)	507 (61.0)		
Master or PHD	20 (5.2)	56 (6.7)	3.500^b^	0.174
Parity (*n*, %)				
1	307 (80.8)	636 (76.5)		
≥2	73 (19.2)	195 (23.5)	2.740^b^	0.101
Fetus number (*n*, %)				
1	350 (92.1)	749 (90.1)		
≥2	30 (7.9)	82 (9.9)	1.209^b^	0.271
Maternal age (years, mean ± std)	30.20 ± 4.77	30.88 ± 4.34	2.447^a^	0.015*
Delivery (*n*, %)				
Natural labor	185 (48.7)	366 (44.0)		
Cesarean section	195 (51.3)	465 (56.0)	2.265^b^	0.132
Complication of pregnancy (*n*, %)				
No	263 (69.2)	628 (75.6)		
Yes	117 (30.8)	203 (24.4)	5.427^b^	0.020*
Household monthly income per capita (*n*, %)				
≤5,000 RMB	34 (8.9)	66 (7.9)		
5,000–10,000 RMB	195 (51.3)	368 (44.3)		
>10,000 RMB	151 (39.7)	397 (47.8)	6.814^b^	0.033*

FD, feeding difficulty; PHD, doctor of philosophy; std, standard deviation; *n*, numbers; %, percentage.

*p < 0.05.

^a^ *t*, independent *t*-test.

^b^ *χ*^2^, chi-square test.

### Prevalence of FD

**Figure 1:**
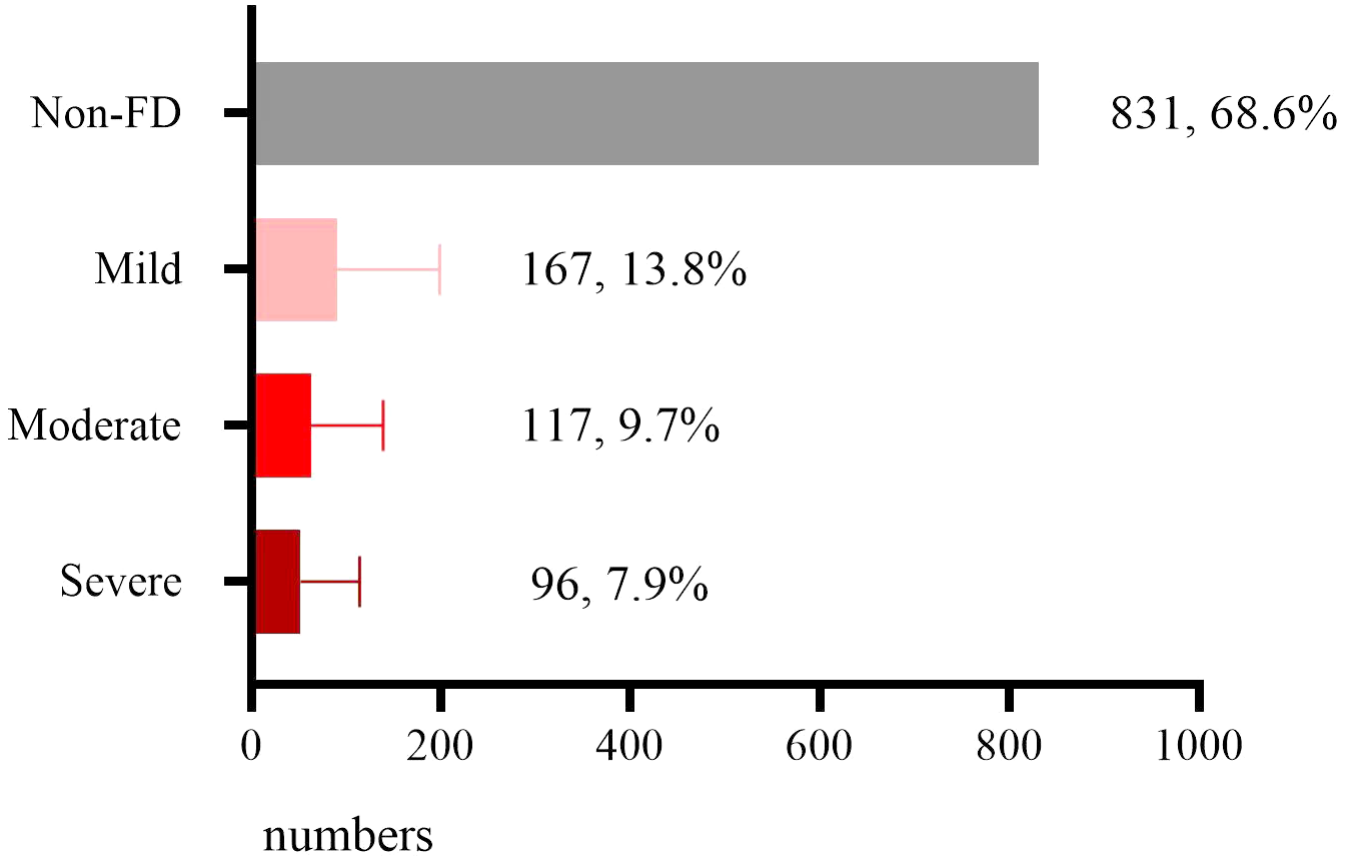
Prevalence of FD in children at self-feeding transition stage.

Among 1,211 valid questionnaires, 380 children were reported as FD with an prevalence of 31.4%, including 167 cases of mild FD (13.8%), 117 moderate (9.7%), and 96 severe (7.9%). Details are illustrated in Figure 1.

### Predictors of FD

Results from chi-squared test employed in the univariate analysis showed 19 potential predictors of FD, including disease at birth, frequent constipation, frequent diarrhea, frequent food allergy under CHILD sub-theme; time of introducing complementary food, food preparing under FOOD sub-theme; anxiety, quarrels, parenting style, time for parenting company under PARENT sub-theme; and observing hunger and satiety signals, training of self-feeding, luring to eat, forcing to eat, allowing playing during mealtime, interacting with child during mealtime, providing fixed table, providing exclusive tableware, quiet environment under FEEDING PRACTICE sub-theme. Detailed results are shown in Table 2.

**Table 2:** Potential predictors associated with FD in the univariate analysis.

Categories	Variables	FD (*n* = 380)	Non-FD (*n* = 831)	*χ* ^2^	*p*
Child	Gestational age (*n*, %)				
	≤37 weeks	74 (19.5)	157 (18.9)		
	>37 weeks	306 (80.5)	674 (81.1)	0.057	0.811
	Weight at birth (*n*, %)				
	<2,500g	91 (23.9)	174 (20.9)		
	≥2,500g	289 (76.1)	657 (79.1)	1.391	0.240
	Disease at birth (*n*, %)				
	Without	338 (88.9)	776 (93.4)		
	Asphyxia	27 (7.1)	32 (3.9)		
	Digestive disease	5 (1.3)	3 (0.4)		
	Endocrine diseases	7 (1.8)	16 (1.9)		
	Cardiovascular disease	3 (0.8)	4 (0.5)	10.262	0.036*
	Frequent constipation (*n*, %)				
	No	308 (81.1)	762 (91.7)		
	Yes	72 (18.9)	69 (8.3)	28.717	0.000***
	Frequent vomiting (*n*, %)				
	No	343 (90.3)	757 (91.1)		
	Yes	37 (9.7)	74 (8.9)	0.217	0.642
	Frequent diarrhea (*n*, %)				
	No	345 (90.8)	803 (96.6)		
	Yes	35 (9.2)	28 (3.4)	18.040	0.000***
	Frequent food allergy (*n*, %)				
	No	307 (80.8)	770 (92.7)		
	Yes	73 (19.2)	61 (7.3)	37.334	0.000***
	Cow's milk protein allergy (*n*, %)				
	No	318 (83.7)	728 (87.6)		
	Yes	62 (16.3)	103 (12.4)	3.407	0.065
Food	Exclusive breastfeeding (*n*, %)				
	No	58 (15.3)	164 (19.7)		
	Yes	322 (84.7)	667 (80.3)	3.483	0.062
	Time of introducing complementary food (*n*, %)				
	<4 month	11 (2.9)	4 (0.5)		
	4–6 month	133 (35.0)	302 (36.3)		
	>6 month	236 (62.1)	525 (63.2)	12.440	0.002**
	Food preparing (*n*, %)				
	No	109 (28.7)	112 (13.5)		
	Yes	271 (71.3)	719 (86.5)	40.416	0.000***
Parent	Anxiety (*n*, %)				
	No	114 (30.0)	576 (69.3)		
	Yes	266 (70.0)	255 (30.7)	164.414	0.000***
	Quarrels (*n*, %)				
	No	229 (60.3)	717 (86.3)		
	Yes	151 (39.7)	114 (13.7)	103.265	0.000***
	Parenting style (*n*, %)				
	Responsive	132 (34.7)	440 (52.9)		
	Controlling	143 (37.6)	302 (36.3)		
	Indulgent	87 (22.9)	63 (7.6)		
	Neglectful	18 (4.7)	26 (3.1)	69.651	0.000***
	Time for parenting company (*n*, %)				
	<4 h/day	86 (22.6)	202 (24.3)		
	4–8 h/day	108 (28.4)	239 (28.8)		
	8–12 h/day	100 (26.3)	152 (18.3)		
	>12 h/day	86 (22.6)	238 (28.6)	11.907	0.008**
Feeding practice	Observing hunger and satiety signals (*n*, %)				
	No	125 (32.9)	143 (17.2)		
	Yes	255 (67.1)	688 (82.8)	37.234	0.000***
	Training of Self-feeding (*n*, %)				
	No	170 (44.7)	258 (31.0)		
	Yes	210 (55.3)	573 (69.0)	21.386	0.000***
	Luring to eat (*n*, %)				
	No	157 (41.3)	648 (78.0)		
	Yes	233 (58.7)	183 (22.0)	157.272	0.000***
	Forcing to eat (*n*, %)				
	No	242 (63.7)	726 (87.4)		
	Yes	138 (36.3)	105 (12.6)	91.154	0.000***
	Allowing playing while eating (*n*, %)				
	No	179 (47.1)	624 (75.1)		
	Yes	201 (52.9)	207 (24.9)	91.412	0.000***
	Interacting with child while eating (*n*, %)				
	No	82 (21.6)	79 (9.5)		
	Yes	298 (78.4)	752 (90.5)	32.968	0.000***
	Between-meal nibbles (*n*, %)				
	No	179 (47.1)	393 (47.3)		
	Yes	201 (52.9)	438 (52.7)	0.004	0.952
	Meal time limit (*n*, %)				
	No	259 (68.2)	586 (70.5)		
	Yes	121 (31.8)	245 (29.5)	0.688	0.407
	Providing exclusive tableware (*n*, %)				
	No	89 (23.4)	102 (12.3)		
	Yes	291 (76.6)	729 (87.7)	24.389	0.000***
	Providing fixed table or chair (*n*, %)				
	No	44 (11.6)	26 (3.1)		
	Yes	336 (88.4)	805 (96.9)	34.188	0.000***
	Quiet environment (*n*, %)				
	No	112 (29.5)	68 (8.2)		
	Yes	268 (70.5)	763 (91.8)	93.407	0.000***
	Eating with parents (*n*, %)				
	No	171 (45.0)	378 (45.5)		
	Yes	209 (55.0)	453 (54.5)	0.025	0.874

FD, feeding difficulty; *χ*^2^, chi-square test; *n*, numbers; %, percentage.

*p < 0.05.

**p < 0.01.

***p < 0.001.

**Figure 2:**
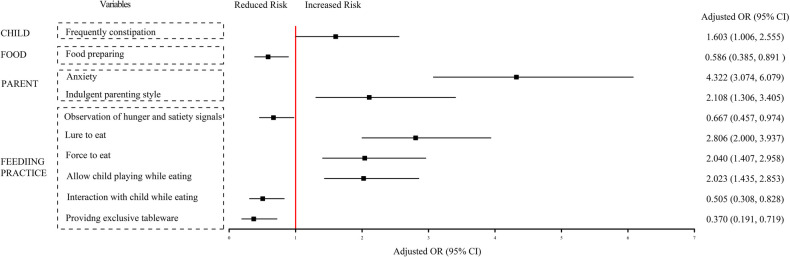
Independent predictors associated with FD in multivariate logistic regression analysis. After corrected with “Maternal age, Complication of pregnancy and Household monthly income per capita” from demographic characteristics data. FD, feeding difficulty; OR, odd ratio; CI, confidence interval.

Results from adjusted binary logistic regression employed in the multivariate analysis selected 10 out of 19 independent preditors of FD. Among them, 6 were risk factors including frequent constipation (*OR* = 1.603, *CI* = 1.006–2.555) from CHILD; anxiety (*OR* = 4.322, *CI* = 3.074–6.079) and indulgent parenting style (*OR* = 2.108, *CI* = 1.306–3.405) from PARENT; luring to eat (*OR* = 2.806, *CI* = 2.000–3.937), forcing to eat (*OR* = 2.040, *CI* = 1.407–2.958), and allowing playing during mealtime (*OR* = 2.023, *CI* = 1.435–2.853) in FEEDING PRACTICE, and 4 of them protective factors including food preparing (*OR* = 0.586, *CI* = 0.385–0.891) in FOOD; observing hunger and satiety signals (*OR* = 0.667, *CI* = 0.457–0.974), interacting with child during mealtime (*OR* = 0.505, *CI* = 0.308–0.828), as well as providing exclusive tableware (*OR* = 0.370, *CI* = 0.191–0.719) in FEEDING PRACTICE. The results are summarized in Table 3 and illustrated in Figure 2.

**Table 3:** Independent predictors associated with FD in multivariate logistic regression analysis.

Category	Variables	*B*	S.E.	Wald	*p*	*OR* (95% CI)
Child	Frequent constipation	0.472	0.238	3.937	0.047*	1.603 (1.006, 2.555)
Food	Food preparing	−0.535	0.214	6.244	0.012*	0.586 (0.385, 0.891)
Parent	Anxiety	1.464	0.174	70.784	0.000***	4.322 (3.074, 6.079)
	Indulgent parenting style	0.746	0.245	9.3.5	0.002**	2.108 (1.306, 3.405)
Feeding practice	Observing hunger and satiety signals	−0.405	0.193	4.386	0.036*	0.667 (0.457, 0.974)
	Luring to eat	1.032	0.173	35.658	0.000***	2.806 (2.000, 3.937)
	Forcing to eat	0.713	0.190	14.146	0.000***	2.040 (1.407, 2.958)
	Allowing playing while eating	0.705	0.175	16.160	0.000***	2.023 (1.435, 2.853)
	Interacting with child while eating	−0.683	0.252	7.336	0.007**	0.505 (0.308, 0.828)
	Providing exclusive tableware	−0.993	0.338	8.629	0.003**	0.370 (0.191, 0.719)

After corrected with “Maternal age, Complication of pregnancy and Household monthly income per capita” from demographic characteristics data. FD, feeding difficulty; OR, odd ratio; CI, confidence interval.

*p < 0.05.

**p < 0.01.

***p < 0.001.

## Discussion

Children are supposed to develop self-feed skills in their first 2 years of life, while 6–24 months of age is the most critical period for children to form appropriate eating behaviors (16). During this stage, young children's eating problems can easily provoke parents' anxiety, and parents faced with FD are more likely to experience stress and helplessness, so professional advice and guidance are in urgent demand among them. Given that, we conducted this multi-center cross-sectional study to explore the prevalence and predictors of FD in young children at this problem-prone stage, in the hope of offering some enlightenment on the prevention of FD.

### Current prevalence of FD in young children worldwide

Recent studies abroad have reported FD prevalence ranging from 25% to 50% in young children (7–9), while among those suffering from growth retardation or diseases such as autism, the prevalence can get as high as 80% (17, 18). In 2013, Zhao et al. (14) carried out an epidemiological survey on FD prevalence in young children aged 6–24 months in 69 maternal and child health care hospitals in China, in which a total of 4,017 participants were investigated and the FD prevalence was 21.4%. In our study, we found an FD prevalence of 31.4% for children aged 6–24 months, which was within the range of previous foreign studies (7–9), but higher than reported in Zhao's et al. (14) study, revealing a possible increasing of FD prevalence in China. However, it is also worth noting that the FD prevalence in young children appears to be rising both in the domestic and abroad. A nationwide prevalence study in USA has reported that the FD prevalence in young children presented an ascending trend over a 5-year period from 2009 to 2014, suggesting that FD has gradually become a common issue in pediatric clinic (19). We reckon this trend may have been caused by a series of multi-factors related to social development, and here we offer a possible speculation that may interpret the underlying mechanism: With a rapid economic growth, the birth rate has gradually decreased while the population education level has been continuously improved. With fewer children in one family, parents tend to contribute more to parenting, and regard child-raising as a more elaborate job. Once meticulous feeding becomes ubiquitous, feeding problems emerge in large numbers. As is the case with China, although the government has recently released the three-child policy, many urban young parents still choose to raise only one child for the time being, and this may lead to parents' extra focus on children's eating problems, which is not conducive to the forming of proper eating behaviors. Nevertheless, what has mainly caused this apparent increasing trend of FD prevalence in young children worldwide is not yet completely determined, due to the heterogeneous definitions and evaluation methods adopted by various studies, and further evidence is needed to prove our theory stated above.

### Independent predictors of FD

Our study included 27 potential predictors grouped by four sub-themes, from which we derived 10 independent predictors of FD, including 4 protective predictors and 6 risk predictors.

### Protective predictors

As one of the protective predictors, food preparing was the only one proved to be an independent predictor of FD from FOOD sub-theme. As for other food-related factors, previous researches have studied whether exclusive breastfeeding and time of introducing complementary food are associated with FD, but have acquired seemingly controversial results. In this study, we did not observe any differences in FD in children who were breastfed. Similarly, De Barse et al. (20) found children who were never breastfed did not differ in selective eating frequency from children breastfed for 6 months or longer. However, it is generally believed that exclusive breastfeeding can reduce the risk of FD, as various flavors of breast milk make exclusive-breastfed children more likely to accept different types of complementary foods (21). Besides, we did not observe any differences in FD in children who were introduced with complementary food <4, 4–6 or >6 month, but Hollis et al. (22) found that complementary food introduction after 6 months could reduce the risk of FD compared with that between 4 and 6 months, whereas De Barse et al. (20) suggested that introduction of vegetables into children's diet before 5 months might contribute to lower FD risk. Due to the lack of sufficient evidence in existing studies to demonstrate the relationship between breastfeeding, complementary feeding time and feeding difficulties, it is recommended that future studies would further confirm this issue through evidence-based methods. In the final adjusted regression, the results showed that among all the food-related potential factors, only food preparing was proved to be an independent protective predictor. Children at self-feeding transition stage are supposed to experience foods with different textures and multiple flavors, and the most effective way to prevent FD is simply for parents to attentatively prepare food for their young children. Practical strategies of food preparing in the purpose of improving children's appetite include: investigating children's food preferences, providing them with food of age-appropriate textures, alternating cooking methods, selecting food with adorable shapes, as well as adding in seasonings properly. Furthermore, frequent exposure to new foods, and allowing touching and playing with all varieties of foods might improve children's acceptance of whatever they are feeding.

In FEEDING PRACTICE, protective predictors include observing hunger and satiety signals, interacting with child during mealtime and providing exclusive tableware, which suggest that accurate capture of hunger and satiety signals, positive parent-child interaction during mealtime, and provision of exclusive tableware can reduce the risk of FD. Similar results were found in research conducted by Atzaba-Poria et al. (23) that more intimate parent-child interaction during mealtime leads to a lower chance of FD. Van Den Engel-Hoek et al. (24) stated in their study that inappropriate eating behavior was reduced proportionally to the children's learning development of utilizing feeding equipment.

**Figure 3:**
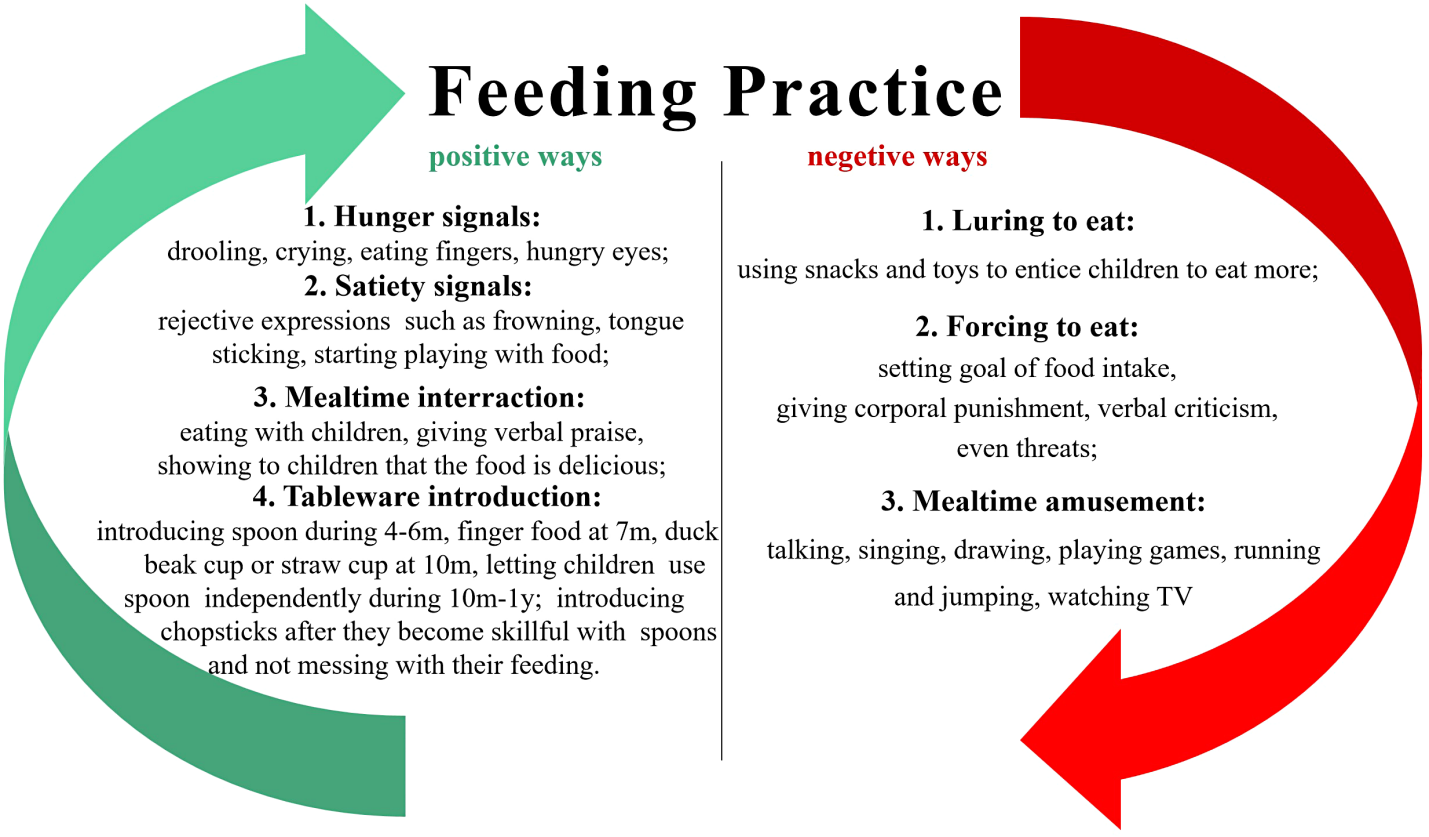
Highlights in feeding practice.

As far as we are concerned, prevention is of much greater importance than treatment, and understanding these protective factors will help child health care clinicians and pediatricians guide parents to prevent FD from young children effectively in their routine work. Thus, we highlight the significance of health education to parents, and here we offer some important feeding practices as showed in Figure 3.

### Risk predictors

We present 6 risk predictors of FD here, with 1 from CHILD, 2 from PARENT and 3 from FEEDING PRACTICE.

Among child-related factors, long-term frequent constipation appears to increase the risk of FD. As far as we know, no similar conclusion has been drawn in relevant studies. One possible explanation is that long-term frequent constipation is related to gastrointestinal dysfunction which causes physical discomfort, making it difficult for parents to feed their children. As for the remaining factors, some believe that premature birth and other diseases may be correlated with FD, probably due to the fact that children under these conditions almost always enjoy more tolerance from their parents, thus developing inappropriate eating behaviors. However, Sanchez et al. (25) found that no difference was shown in eating behaviors at the age of 3 years between preterm and full-term infants, which to some degree is consistent with our results that preterm birth showed an inadequate relationship with FD. Whether premature birth and other disease conditions are risk predictors of FD are still undefined according to existing literature.

Normally it is parents who decide when, where, what and how much their children are fed, so parents have a crucial impact on children's eating behaviors. Consistent with most of the previous studies (3, 26–28), our results suggested that anxiety is the main risk predictor of FD among parents-related factors. In fact, more than 50% of parents in our study believed that their children had various feeding problems (2, 3), and more than one third of parents expressed concern about feeding processes (28). Anxiety seems to be an unavoidable problem in child-raising. Although no studies have yet been able to construct a complete model describing the complex relationship between parental anxiety and FD, reasonable interpretations and explanations about it have been made. For instance, the correlation between parental anxiety and FD in children was well illustrated in a study which suggested that children's food aversion and refusal behaviors could be affected by tense parent-child interactions during mealtime due to parental anxiety (26). Moreover, Lee et al. (28) found that anxious parents who tend to excessively focus on their children's diet and eating behaviors are prone to adverse feeding strategies such as forced feeding, which makes their children resist eating and thus complete the vicious circle of parental anxiety and FD. In cases where parents are under extreme anxiety, detailed guidance from pediatricians is very necessary. Clarifying the pathogeny of FD and offering specific suggestions can be of paramount importance to help parents reduce anxiety, and it also turn out an effective way to try to shifting their attention from temporal figures such as children's weight gains and food intake to something of practical significance (29). For instance, pediatricians can advise parents to start from enhancing children's activities or preparing food.

Another parent-related risk predictor of FD revealed by our study is indulgent parenting style. Previous literature has identified that 81.2% of parents reported non-responsive feeding style (28), one of the major risk predictors of FD (30, 31), while our study suggested indulgent parenting style to be the most adverse. In China, most children born between 1980s and 1990s are the only children in their families. Now these children have reached childbearing age and their parents have approached retirement, which generates the special intergenerational structure of “4 + 2 + 1” in most urban families. Being the only one of the third generation in a family, the young grandchild at the bottom rank of this inverted triangle family structure is naturally favored since born, and in many cases raised by grandparents rather than its own parents. Thus, indulgent feeding style is ubiquitous in these families. When treating these young parents with their own only child from this special family structure, timely and correct guidance from pediatricians on their feeding practice is most crucial, because it is they who should be encouraged to play a leading role in the parenting process instead of their child's grandparents.

Our study also found that factors including luring to eat, forcing to eat and allowing playing during mealtime are also risk predictors of FD from the FEEDING PRACTICE sub-theme. Luring to eat and allowing playing during mealtime are seemingly effective ways to increase children's food intake by diverting their attention, such as inducing children to eat more food using snacks, allowing children to watch animations while feeding, etc. Typical performances can be seen in Figure 3. However, since children's adulthood eating habits can be adversely impacted, these approaches are not recommended by us. It's also worth noting that it is very easy to cause airway foreign bodies by making children laugh during mealtime, and pediatricians are obliged to correct inappropriate and dangerous feeding practice of parents. Besides, forcing to eat seems to be a common practice in China, where grandparents or parents are excessively concerned about children's food intake, and those suffering from serious parental anxiety even do not allow leftovers (14). This kind of compulsive behavior can make children resistant to eating. The more proper way to do this is to limit the mealtime within 25–30 min (7, 8), and what parents need to do most is to create a harmonious atmosphere for eating and to provide children with age-fit food as well as exclusive tableware. As for how much to be fed, it should be and will be dependent on children themselves only.

## Strengths and limitations

6–24 months of age is the most critical period for children to form appropriate eating behaviors. This study is one of the few that focuses on exploring the prevalence and potential predictors of FD in young children during this period. Both protective and risk factors from comprehensive perspectives have been analyzed. Our results serve as enlightenment and provide strong support for child health care clinicians and pediatricians to guide parents on how to prevent FD. Admittedly, limitations existed as follows. Firstly, an overview concerning our research objects (FD parents and children) is not available in rural areas since research centers are all located in urban areas, regional restrictions thus were inevitable. Secondly, this study involves five research centers. Although these five hospitals are located in the same city, and the response effective rate is similar between each research center, there may be some differences in the demographic characteristics of patients between each research site, which was not discussed in depth in this study. Finally, the measurement in this study as Potential Predictors of FD Questionnaire was developed by the researcher, and have not been tested for validity. Future study are recommend to develop a well-structured and valid scale to assess the associated factors of FD.

## Conclusions

This is a multi-center cross-sectional study on the prevalence and predictors of FD in children at self-feeding transition stage in China. The results showed that the prevalence of FD in young children was 31.4% presenting an ascending trend with time, and meticulous feeding lead to parental excessive concern about children's feeding issues is the main reason behind this. We identified 10 independent predictors of FD, including food preparing, observing hunger and satiety signals, interacting with child during mealtime, providing exclusive tableware as protective factors, while frequent constipation, parental anxiety, indulgent parenting style, luring to eat, forcing to eat, allowing playing during mealtime as risk factors. Our study advocates that child health care clinicians and pediatricians ought to attach more attention to the prevention of FD in young children. They are obliged to correct improper feeding strategies of caregivers in time, and ensure that they feed their children correctly under guidance. Most importantly, feeding guidance keeping up with the times needs continuous developing and updating.

## Data Availability

The original contributions presented in the study are included in the article/Supplementary Material, further inquiries can be directed to the corresponding author.

## Keynotes

• There appeared to be an increasing trend of FD prevalence with 31.4% in China which might be linked with social and economic development.
• 6 risk factors and 4 protective factors were determined associated with FD in children at self-feeding transition stage.
