# Supplementary material for: The prevalence and predictors of feeding difficulties in children at self-feeding transition stage
Source: Front Pediatr. 2023 Jul 10;11:1175927. doi: 10.3389/fped.2023.1175927 (PMC10363730; doi:10.3389/fped.2023.1175927)
Supplement: Supplementary file 1 [file Presentation1.pdf]

Potential Predictors of FD Questionnaire

CHILDREN

1. Your child was born at a gestational age of________

2. Your child was born at a birthweight of________

3. Did your child have any illnesses at birth__________, if yes, please list specific disease information________

4. Has your child ever been diagnosed with cow milk protein allergy by doctors? ________

5. Does your child frequently vomit milk before adding complementary foods (with an average of ⩾3 times a day and a vomiting volume of ⩾20ml milk each time)? ________

6. Does your child often experience constipation (at least once every 3 days) since the addition of complementary foods?________

7. Has your child experienced frequent diarrhea since the addition of complementary foods (average of ⩾5 times per day)?________

8. Does your child often experience allergies due to the addition of new foods since the addition of complementary foods? (such as bleeding, eczema, diarrhea, etc., with ⩾ 3 occurrences)? ________

FOOD

1. Do you breastfeed? (Refers to feeding infants solely from the mother's milk during lactation)________

2. When does your child start adding complementary foods?________

3. Have you considered the diversity of ingredients, texture, and cooking methods when prepare complementary foods for your child?________

PARENT

1. The average time you spend company with your child every day (excluding sleeping time) is?________

2. Do you often feel anxious about your child's feeding issues?________

3. Do you often argue with other caregivers in your family about child's feeding issues?_____

4. Which type of feeding style (responsive-responding to child's cues, controlling-overriding child's cues, indulgent-catering to child's desires, neglectful-unaware of child's cues) do you think you have? ________

FEEDING PRACTICE

1. Do you often intentionally observe the child's signs of hunger and satiety?
2. Do you always try to let child eat on their own during meals? (such as catching or feeding oneself using a spoon)
3. Do you always chase or tease your child to lure to feed during meals?
4. Do you always force your child to finish the food?
5. Do you allow child to engage in recreational activities while eating? (such as watching TV or playing with toys)
6. Do you have parent-child interaction with child during meals? (such as verbal encouragement and eye contact)
7. Do you allow child to consume snacks or drinks between meals? (Including specially made snacks and freshly squeezed fruit juice)
8. Do you limit child's dining time to no more than 25 minutes?
9. Do you provide exclusive tableware for child during meals?
10. Do you provide a fixed dining table or chair for your child during meals?
11. Do you provide a quiet and harmonious dining environment for child during meals?
12. Do you eat with your child together?

The Montreal Children's Hospital Feeding Scale

1. How do you find mealtimes with your child?
2. How worried are you about your child's eating?
3. How much appetite (hunger) does your child have?
4. When does your child start refusing to eat during mealtimes?
5. How long do mealtimes take for your child (in minutes)?
6. How does your child behave during mealtimes?
7. Does your child gag or spit or vomit with certain types of food?
8. Does your child hold food in his/her mouth without swallowing it?
9. Do you have to follow your child around or use distractions (toys, TV) so that your child will eat?
10. Do you have to force your child to eat or drink?
11. How are your child's chewing (or sucking) abilities?
12. How do you find your child's growth?
13. How does your child's feeding influence your relationship with him/her?
14. How does your child's feeding influence your family relationships?
